# Blue laser inferior turbinate reduction for medically refractory chronic rhinitis: a retrospective analysis of clinical outcomes and optimal energy settings

**DOI:** 10.1007/s10103-026-04835-2

**Published:** 2026-03-07

**Authors:** Cheng-Jung Wu, Sheng-Yu Wu, Cheng-Yu Tsai, Jinn-Moon Yang, Lok-Yee Joyce Li

**Affiliations:** 1 Department of Otolaryngology, School of Medicine, College of Medicine, Taipei Medical University, Taipei, Taiwan; 2 Department of Otolaryngology, Shuang Ho Hospital, Taipei Medical University, New Taipei, Taiwan; 3 Ph.D. Degree Program of Biomedical Science and Engineering, National Yang Ming Chiao Tung University, Hsinchu, Taiwan; 4 Department of Industrial Engineering and Industrial Management, National Tsing Hua University, Hsinchu, Taiwan; 5 Department of Civil and Environmental Engineering, Imperial College London, London, UK; 6 Institute of Bioinformatics and Systems Biology, National Yang Ming Chiao Tung University, Hsinchu, Taiwan; 7 Department of Biological Science and Technology, National Yang Ming Chiao Tung University, Hsinchu, Taiwan; 8 Department of Environmental and Occupational Medicine, National Taiwan University Hospital Hsin-Chu Branch, Hsinchu, Taiwan

**Keywords:** Rhinitis, Radiofrequency, Turbinate reduction, Blue laser

## Abstract

This study aims to evaluate the clinical efficacy of 445 nm blue laser inferior turbinate reduction (BITR) and establish optimal energy parameters for patients with chronic rhinitis (CR) refractory to pharmacotherapy. This was a retrospective cohort study of adult patients with medically refractory CR, a Nasal Obstruction Symptom Evaluation (NOSE) score ≥ 6, and a nasal congestion score ≥ 2. Turbinate hypertrophy was graded using the Camacho classification. Patients were stratified into Group A (Grade 3) and Group B (Grade 4). Primary endpoints were changes in NOSE scores and 12-hour Reflective Total Nasal Symptom Score (12-h rTNSS) at 1, 3, and 6 months. At 6 months, laser energy settings were analyzed in the top 50% of responders to determine optimal settings. Eighty-four patients were analyzed. At 6 months, NOSE scores improved by a mean of 91.1% in Group A and 88.0% in Group B (*p* < 0.001 for both). At 3 months, the 12-h rTNSS improved by 58.2% and 54.9%, respectively (*p* < 0.001 for both). In the top 50% of responders, the optimal mean energy delivered was 219 J (anterior) and 51 J (middle) for Group A, and 332 J (anterior) and 60 J (middle) for Group B. Postoperative complications were minimal. BITR is a safe and effective office-based procedure for medically refractory CR. To optimize outcomes, we recommend energy settings of approximately 219 J (anterior) and 51 J (middle) for moderate hypertrophy, and 332 J (anterior) and 60 J (middle) for severe hypertrophy.

## Introduction

Chronic rhinitis (CR) is a significant global health concern, impacting patient quality of life through sleep disruption and reduced cognitive function. Its etiology is heterogeneous, encompassing allergic and non-allergic phenotypes, but its pathophysiology involves persistent inflammation of the nasal mucosa, leading to chronic edema, submucosal collagen deposition, and ultimately, irreversible inferior turbinate hypertrophy (ITH). In fact, inferior turbinate hypertrophy anatomically explains the refractories of nasal congestion. Surgical intervention becomes necessary when corticosteroids and other conservative treatments are no longer effective at managing patients with the above-mentioned symptoms. Surgical strategies evolve from invasive approaches to functional conservative manners, intended to preserve the overlying mucosa. Conventional treatment options include submucosal resection and microdebrider-assisted inferior turbinate rhinoplasty. As surgical techniques innovate, minimally invasive and energy-based devices have become a new clinical standard method. One of the examples includes radiofrequency ablation, RFA, which was shown to improve Nasal Obstruction Symptom Evaluation (NOSE) score by 76% [1].

Laser technology, such as conventional diode laser at 980 nm, is effective that symptomatic relief can be seen in 86% of patients [2]. Yet, crusts formation and squamous metaplasia of the respiratory epithelium [3] are not uncommon side effects. In fact, significant differences in the degree of thermal damage have been seen between different laser energy systems; conventional diode lasers bring about long-lasting crusts for more than 2 months, while most patients who received 445 nm blue laser treatment have crusts which last less than one month. Other laser systems have different drawbacks respectively. For example, the effectiveness of Holmium: YAG (Ho: YAG) laser declines over time; patient satisfaction decreased from 89% at 6 months post-surgery to 52% at 16 months [4]. In addition, highly water absorptive laser systems, such as CO₂ and Erbium: YAG, were designed to perform superficial tissue ablation, but uncontrollable thermal distribution in deeper areas poses risks to submucosal reduction surgeries [5].

The 445 nm blue laser represents a technological advancement. Its wavelength has a high absorption coefficient in hemoglobin and melanin but negligible absorption in water. Photoangiolytic effect is achieved when energy is selectively absorbed in the vessel-rich submucosal stroma. Such targeted energy transmission not only minimizes the thermal damage of the surrounding mucosal lining, but also efficiently attains tissue debulking and focal hemostasis by means of precise vaporization and solidification. Albeit the above-mentioned biophysical privilege, limited studies about the application of 445 nm blue laser on managing ITH were available. The involvement of ITH in standardized treatment regimen is rather rare. Existing studies often report power (Watts) but not clear, outcome-correlated energy dosimetry guidelines in Joules, leading to outcome variability. Therefore, this study aims to: (1) evaluate the clinical outcomes of 445 nm BLTR in patients with medically refractory CR, and (2) perform a dosimetric analysis to identify optimal energy parameters based on the anatomical severity of ITH, providing the first data-driven evidence-based guidelines for this procedure.

## Materials and methods

### Study design and patient population

This retrospective cohort study was approved by the institutional review board (TMU-JIRB No. N202509008), and the requirement for informed consent was waived. The study included adult patients treated for medically refractory CR between September 2024 and March 2025. Inclusion criteria were: (1) treatment failure after at least three months of medical therapy (intranasal corticosteroids with or without oral antihistamines); (2) baseline NOSE score ≥ 6 [6]; (3) baseline nasal congestion sub-score ≥ 2; and (4) Grade 3 (51–75% obstruction) or Grade 4 (75–100%) turbinate hypertrophy according to the Camacho classification [7].

While the underlying etiology of CR (e.g., allergic or non-allergic) was noted in patient history, it was not an exclusion criterion for this study, which focused on the anatomical outcome of turbinate reduction. As this was a retrospective study, specific diagnostic data (e.g., skin prick tests or serum-specific IgE levels) to definitively sub-classify the etiology for every patient (allergic versus non-allergic rhinitis ) were not systematically available in all patient charts. Patients with significant septal deviation, chronic rhinosinusitis, previous nasal surgery, or rhinitis medicamentosa were excluded. Rhinitis medicamentosa was excluded to avoid intraoperative bleeding risks associated with severe inflammation and to eliminate the confounding effect of vasoconstrictors on baseline turbinate grading.

### Preoperative assessment and stratification

All patients underwent a standardized evaluation, including clinical history and nasal endoscopy. Symptom severity was quantified using two validated patient-reported outcome measures.

- Nasal Obstruction Symptom Evaluation (NOSE) scale: A 5-item questionnaire assessing nasal obstruction severity and its quality-of-life impact, with a total score from 0 to 100 [6].
- **12-hour reflective Total Nasal Symptom Score (rTNSS)**: A 4-item instrument scoring nasal congestion, rhinorrhea, itching, and sneezing, with a total score from 0 to 12 [1].

ITH was graded using the Camacho classification system [7]. Patients were stratified into Group A (Grade 3) and Group B (Grade 4).

### The 445 nm blue laser surgical technique

Surgeries were performed by one surgeon in an out-patient setting. Under local anesthesia, The WOLF TruBlue 445 nm Laser equipment with 400 μm diameter blue laser fiber that outputs 445 nm wavelength laser energy was applied to each patient. The use of vasoconstrictors (such as oxymetazoline) was strictly prohibited amid the operation, ensuring accurate evaluation of the debulking effect of laser energy. All recruited patients received comprehensive pre-operative assessment to rule out any contraindication of the surgery. In addition, patients were required to take rest and be observed for at least 30 min, in case any immediate complications, like bleeding, develop.

The power setting was determined based on the manufacturer’s recommendations for submucosal tissue coagulation and ablation, as well as preliminary institutional experience aimed at achieving visible tissue blanching without causing excessive charring. A power of 2.5 W in a super-pulse wave mode (100 ms on, 100 ms off) was found to provide optimal balance between efficacy and safety. Under the guidance of endoscope, the laser fiber was applied on the submucosal layer of the inferior turbinate. Gentle energy was transferred in a flo and back manner to create several submucosal tunnels, especially in the first third and the middle third areas. The ideal range of surgery was evaluated visually by observing the degree of tissue blanching and manually by palpating volume reduction. Total energy delivered in the above-mentioned process was continuously and meticulously recorded. The visual endpoint was tissue blanching and palpable volume reduction. Total energy delivered (Joules) was meticulously recorded. The laser safety operating procedures were strictly followed during the surgery. Surgeon and all nurses wore specialized protective goggles compatible with the laser wavelength and used a smoke extraction system to remove the smoke generated during laser surgery, which ensured good surgical field visibility and surgical safety. All patients meet standard surgical safety requirements, including preoperative assessment to rule out contraindications, postoperative observation for at least 30 min to monitor for immediate complications (such as bleeding), and clear postoperative care instructions. Patients also receive 24-hour contact information from the surgical team for immediate postoperative consultation. Postoperatively, patients are advised to avoid strenuous activity, nose blowing, and contact with any irritants that may irritate the nasal mucosa for two weeks.

### Outcome measures and follow-up protocol

Postoperative follow-up was conducted at 1, 3, and 6 months. Primary endpoints were the change from baseline in NOSE and 12-h rTNSS scores. The secondary endpoint was the determination of optimal energy parameters, defined as the mean energy delivered to the top 50% of responders (greatest % improvement in NOSE score at 6 months) within each group. Adverse events were systematically recorded.

### Statistical analysis

Data were analyzed using SPSS Statistics (Version 28.0). Descriptive statistics were used for baseline characteristics. The Wilcoxon signed-rank test was used for preoperative and postoperative comparisons of NOSE and rTNSS scores within groups. The Mann-Whitney U test was used for between-group comparisons. A p-value < 0.05 was considered statistically significant.

## Results

### Patient characteristics

Eighty-four patients were included (62 male, 73.8%; mean age 36.2 ± 11.5 years). Group A included 38 patients and Group B included 46. Baseline demographic and clinical characteristics were statistically comparable between groups (Table 1).

**Table 1:** Baseline demographics and clinical characteristics of the study cohort

Characteristic	Group A (Grade 3, *n* = 38)	Group B (Grade 4, *n* = 46)	Total (*n* = 84)	*P*-value
Age (years, mean ± SD)	35.5 ± 10.9	36.8 ± 12.1	36.2 ± 11.5	0.64
Sex (Male, n (%))	27 (71.1)	35 (76.1)	62 (73.8)	0.59
Baseline NOSE Score (mean ± SD)	65.2 ± 10.5	68.5 ± 9.8	67.0 ± 10.2	0.15
Baseline rTNSS (mean ± SD)	7.9 ± 1.8	8.2 ± 1.6	8.1 ± 1.7	0.41

### Clinical efficacy and symptom improvement

Both groups showed statistically significant and clinically improvements in both primary outcomes at all follow-up points. At 6 months, the mean NOSE score in Group A decreased from a baseline of 78.2 to 7.1 (90.9% improvement), and in Group B from 81.5 to 11.6 (85.8% reduction) (*p* < 0.001 for both). Similarly, the mean 12-h rTNSS at 6 months improved by 68.0% in Group A and 64.8% in Group B (*p* < 0.001 for both). Longitudinal data are presented in Table 2.

**Table 2:** Longitudinal analysis of NOSE and rTNSS scores at Baseline, 1, 3, and 6 months

Outcome Measure	Time Point	Group A (mean ± SD)	% Improvement	Group B (mean ± SD)	% Improvement
NOSE Score	Baseline	68.2 ± 10.5	-	71.5 ± 9.8	-
	1 Month	15.1 ± 8.2∗	77.9%	18.3 ± 9.5∗	74.4%
	3 Months	9.3 ± 6.5∗	86.4%	13.5 ± 7.9∗	81.1%
	6 Months	6.1 ± 4.9∗	91.1%	8.6 ± 7.1∗	88.0%
rTNSS	Baseline	7.9 ± 1.8		8.2 ± 1.6	
	1 Month	4.1 ± 2.1∗	48.1%	4.5 ± 2.0∗	45.1%
	3 Months	3.3 ± 1.9∗	58.2%	3.7 ± 1.8∗	54.9%
	6 Months	4.3 ± 1.7∗	45.6%	4.7 ± 1.7∗	42.7%

*p* < 0.001 for change from baseline within each group (Wilcoxon signed-rank test)

### Dosimetric analysis and optimal energy settings

Total energy delivered was commensurate with tissue volume, with Group B receiving significantly more energy. In Group A, mean energy was 231 J (anterior) and 62 J (middle). In Group B, mean energies were 329 J and 78 J, respectively. Analysis of the top 50% of responders yielded the recommended optimal energy settings (Table 3).

**Table 3:** Laser energy parameters and recommended optimal settings by hypertrophy grade

Hypertrophy Grade	Anatomical Location Inferior turbinate (IT)	Mean Energy Delivered (All Patients, J)	Optimal Energy Setting (Top 50% Responders, J)
Grade 3	Anterior 1/3 IT	231	219
	Middle 1/3 IT	62	51
Grade 4	Anterior 1/3 IT	329	332
	Middle 1/3 IT	72	60

### Safety and tolerability

The BITR procedure was well-tolerated with minimal postoperative morbidity. Mild crusting was observed in 83% of patients at 1 week, decreasing to 9.5% at one month and resolving in all patients by three months **(**Table 4**)**. No significant postoperative epistaxis, mucosal edema, or persistent pain was recorded. Minor synechiae formation occurred in one patient from each group and was resolved in-office.

**Table 4:** Showing number of patients having complication in 1 week, 1 month, 3 month and 6 month

complications	1 week postoperatiive	1 month postoperatiive	3 month postoperatiive	6 month postoperatiive
Bloody nasal discharge	A: 0 (0%)	A: 0 (0%)	A: 0 (0%)	A: 0 (0%)
	B: 1 (2.1%)	B: 0 (0%)	B: 0 (0%)	B: 0 (0%)
Pain	A: 3 (7.8%)	A: 0 (0%)	A: 0 (0%)	A: 0 (0%)
	B: 4 (8.6%)	B: 0 (0%)	B: 0 (0%)	B: 0 (0%)
Crusting	A: 29 (76.3%)	A: 3 (5.2%)	A: 0 (0%)	A: 0 (0%)
	B: 41 (89.1%)	B: 3 (6.5%)	B: 0 (0%)	B: 0 (0%)
Synechiae	A: 0 (0%)	A: 1 (2.6%)	A: 0 (0%)	A: 0 (0%)
	B: 0 (0%)	B: 1 (2.1%)	B: 0 (0%)	B: 0 (0%)
Mucosal Edema	A: 0 (0%)	A: 0 (0%)	A: 0 (0%)	A: 0 (0%)
	B: 0 (0%)	B: 0 (0%)	B: 0 (0%)	B: 0 (0%)

## Discussion

This study provides compelling evidence that 445 nm BLTR is a safe, well-tolerated, and effective treatment for patients with medically refractory CR secondary to ITH. The procedure yields statistically significant and clinically improvements in nasal obstruction and rhinitis symptoms, durable at 6 months, wherein the NOSE score showed continuous improvement through the sixth month. Crucially, this is the first investigation to establish evidence-based energy dosimetry guidelines tailored to the anatomical severity of ITH, a critical step toward standardizing the procedure and potentially improving its predictability.

In our study, profound improvement up to 88–91% of the Nasal Obstruction Symptom Evaluation (NOSE) Scale was observed, which seems to have surpassed the therapeutic effects of other treatment options. To our knowledge, radiofrequency ablation improved NOSE scale by 76%, while diode laser reached 86.7% improvement in NOSE scale. With its unique biophysical features, 445 nm wavelength delivers outstanding therapeutic effect. Its high hemoglobin absorption rate allows tissue ablation targeted at submucosal vascular sinusoids. Such targeted approach contrasts with the non-selective thermal damage and associated scar formation unavoidably brought about by bipolar laser. Blue light laser possesses precise photoangiolysis, which can improve surgical safety, promote rapid tissue repair, and maintain stable and long-lasting volume reduction effects. These therapeutic benefits are supported by observations of postoperative complications; in this study, 83% of patients experienced minor crusting within the first week post-surgery, while only 9.5% of patients still showed visible crusting at one month post-surgery.

Furthermore, this study indicates that in addition to improving nasal congestion symptoms, 445 nm blue laser inferior turbinate reduction (BITR) also significantly improved the reflective Total Nasal Symptom Score (rTNSS), with an improvement rate of 54% to 58%. These results demonstrate a significant relief of allergy-related symptoms (such as runny nose, sneezing, and nasal itching), thereby improving patients’ quality of life. In contrast, existing studies have shown that radiofrequency ablation (RFA) often needs to be combined with posterior nasal nerve neurolysis (PNNN) to achieve a more ideal symptom control effect [8, 9]. Although posterior nasal nerve neurolysis or vidian neurectomy has been described in the literature for the treatment of refractory rhinitis, these surgeries are technically demanding and carry certain risks.

In this study, BITR demonstrated broad therapeutic effects, which are presumably related to controllable submucosal thermoregulation. This effect can induce fibrotic responses and partial denervation of secretory motor nerves, mimicking the functional benefits of neurectomy through a minimally invasive approach, thereby achieving symptom improvement.

This explains the significant improvement in rTNSS noted in our cohort. Similar phenomenon was noted in histological change associated with diode laser as reported in previous studies [10, 11]. Therefore, BITR is believed to be an uncomplicated yet comprehensive surgical method. However, the current study focused solely on turbinate volume reduction without specific ablation of the posterior nasal nerve. This preservation of neural innervation might explain the slight, albeit statistically insignificant, rebound in rTNSS scores observed at the sixth month compared to the third month. While combined PNN procedures are described in literature for RFA, BITR alone demonstrated sustainable efficacy in this cohort.

Previously published studies discussed energy parameters, such as power (unit: Watt), laser modes (super-pulse, single pulse, continuous wave mode), etc. To our knowledge, this study is the first investigation which constructed evidence-based laser energy (unit: Joules) guidelines (Table 3). The energy database derived from our innovative study builds a cornerstone in constructing scientific framework and standards. In terms of surgical training, this guideline shortens surgeons’ learning curve in managing blue laser techniques. In terms of clinical practice, it further enhances safety by minimizing risk of undertreatment or over treatment (with complications like atrophic rhinitis and empty nose syndrome).

This study has several limitations, including retrospective and non-comparative design, as well as lack of histological analysis. First, due to the retrospective design, our evaluation relied primarily on patient-reported outcome measures (NOSE and rTNSS score) rather than objective functional tests such as acoustic rhinometry or rhinomanometry. Relying on patient-reported outcome measures may be prone to recall bias. Although the 6-month follow up period gave insight to the long-lasting therapeutic effect, longer follow up duration up to one year is advised to validate possible attenuation of laser energy. While patient-reported outcome measures are validated tools that directly reflect patient quality of life, the inclusion of objective airway resistance data in future studies would further corroborate the structural changes associated with symptom relief. Second, the optimal energy settings proposed in this study were derived from a retrospective analysis of the top 50% of responders. While this data-driven approach provides a valuable preliminary guideline for surgeons, these parameters represent an exploratory finding. Future prospective, randomized controlled trials are necessary to validate these settings and establish their generalizability across broader patient populations. Third, regarding the differentiation of rhinitis phenotypes, we were unable to strictly stratify patients into allergic (AR) and vasomotor (VR) groups due to the lack of systematic immunologic data (e.g., specific IgE) in this retrospective cohort. However, it must be emphasized that the inclusion criteria for this study were for patients who were “medically refractory,” meaning they had not achieved sufficient symptom improvement after at least three months of conservative drug treatment (including intranasal steroids). Significant improvement in the reactive global nasal symptom score (rTNSS; assessment items include sneezing, nasal itching, and runny nose) indicates that blue laser volume reduction of the inferior turbinate (BITR) is effective even in patients with allergic symptoms. This therapeutic effect may be related to the reduction of reactive mucosal surface area, and may also be partly attributed to the denervation effect it causes.

Future prospective studies with immunologic stratification are warranted to further elucidate the long-term interaction between laser reduction and medical management for specific rhinitis subtypes.

## Conclusion

445 nm blue laser inferior turbinate reduction serves as a safe, minimally invasive and efficient surgical approach to help patients suffering from medically refractory chronic rhinitis. This surgical innovation attains significant and sustainable resolution of nasal obstruction and associated symptoms. Postoperative morbidity is rare. The dosimetric guidelines established in this study represent a key advance in standardizing this procedure, allowing surgeons to provide comprehensive disease management under an evidence-based framework. Based on these findings, BITR represents a promising technique in the surgical armamentarium for the treatment of chronic nasal obstruction. Based on these findings, BITR represents a promising technique in the surgical armamentarium for the treatment of chronic nasal obstruction.

## Data Availability

The data presented in this study are available on reasonable request from the corresponding author.
